# Clinical Features of Liver Injury Induced by Immune Checkpoint Inhibitors in Japanese Patients

**DOI:** 10.1155/2019/6391712

**Published:** 2019-12-17

**Authors:** Koji Imoto, Motoyuki Kohjima, Tomonobu Hioki, Tomoyuki Kurashige, Miho Kurokawa, Shigeki Tashiro, Hideo Suzuki, Akifumi Kuwano, Masatake Tanaka, Seiji Okada, Masaki Kato, Yoshihiro Ogawa

**Affiliations:** ^1^Department of Medicine and Bioregulatory Science, Graduate School of Medical Sciences, Kyushu University, 3-1-1 Maidashi, Higashi-ku, Fukuoka 812-8582, Japan; ^2^Division of Pathophysiology Medical Institute of Bioregulation, Kyushu University, 3-1-1 Maidashi, Higashi-ku, Fukuoka 812-8582, Japan

## Abstract

**Aim:**

Immune checkpoint inhibitors (ICIs) have improved the survival rate of patients carrying various malignant neoplasms. Despite their efficacy, ICIs occasionally induce liver injury as an immune-related adverse event (irAE). This study aimed to reveal the clinical features of the hepatic irAE in Japanese patients.

**Methods:**

Among 387 patients treated with ICIs, those who developed drug-induced liver injury were investigated. We also describe the histological findings and clinical courses of four patients with hepatic irAE who underwent liver biopsy.

**Results:**

Among the 56 patients with all-grade liver injury, only 11 (19.6%) showed hepatocellular-type liver injury, which resembled autoimmune hepatitis. Thirty-four patients (60.7%) developed cholestatic or mixed-type liver injury, although only one patient showed abnormal image findings in the bile duct. Most patients with grade ≤2 liver injury improved spontaneously, while two patients with biliary dysfunction required ursodeoxycholic acid or prednisolone. Among eight patients with grade ≥3 liver injury, three required no immunosuppressants and five were treated with prednisolone (three of five patients required other types of immunosuppressants). Four patients in the case series showed diverse clinical features in terms of hepatotoxic pattern, symptoms, and the interval time between the initiation of immunotherapy and the onset of the hepatic irAE.

**Conclusions:**

Our findings suggest that ICIs could cause microscopic biliary disorder without any abnormal image finding. Because the hepatic irAE presents diverse clinical features, liver biopsy is recommended to provide appropriate treatments.

## 1. Introduction

Some kinds of cancers escape the host immune system by “immune checkpoint” pathways, which potentiate cancer cell survival [1]. Immune checkpoint inhibitors (ICIs) block the immune checkpoint pathways and re-activate the T-cell responses towards cancer cells. ICIs have improved the survival rate of patients carrying various tumor [2, 3]. Nivolumab and pembrolizumab recognize programmed cell death 1 (PD-1), which is expressed on the cell surface of T lymphocytes, and block the interaction between PD-1 and programmed death ligand (PDL)-1 and -2, which are expressed on cancer cells [4, 5]. Atezolizumab and durvalumab block PD-L [6, 7] and ipilimumab targets cytotoxic T lymphocyte antigen 4 (CTLA-4) on the cell surface of T lymphocytes [8]. The blockage of this ligand-receptor interaction inhibits the inactivation of T lymphocytes and regains the anticancer effects. The clinical benefits of ICIs can be disturbed by the immune-related adverse events (irAEs) caused by the imbalance of the immune system induced by ICIs [9]. The incidence of the all-grade hepatic irAE with anti-PD-1 monoclonal antibodies (mAbs), anti-CTLA-4 mAb, and their combination therapy is in the range of 1% –3% [10, 11], 3%–9% [12], and 18% [13], respectively. The mechanism of the hepatic irAE is presumed to be similar to autoimmune hepatitis, although it is not fully understood [14]. The first-line drug for hepatic irAE treatment is corticosteroids, and mycophenolate mofetil is considered in steroid-resistant cases [15]. Understanding the details of the hepatic irAE is quite important for optimizing patient management because the long-term administration of immunosuppressants might result in significant treatment-related complications. In this study, we evaluated the clinical and histopathological features of the hepatic irAE experienced in our hospital and treatment strategy. We also describe the histology and clinical course of four patients with hepatic irAE.

## 2. Patients and Methods

### 2.1. Patients

**Figure 1:**
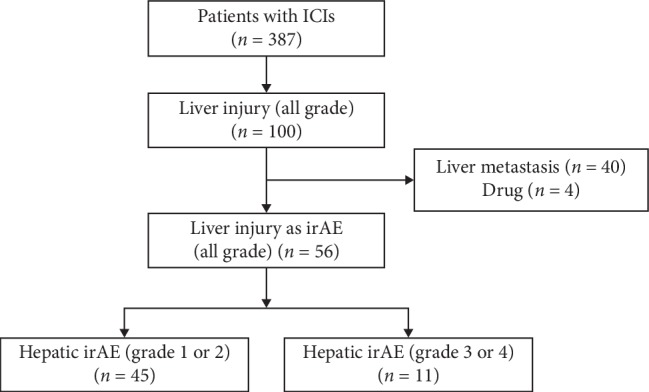
Flowchart of the 387 patients with hepatic irAE included in this study. ICIs, immune checkpoint inhibitors; irAE, immune-related adverse event.

From January 2014 to February 2019, 387 patients were treated with ICIs in our hospital and enrolled retrospectively in this observational study. This study was carried out in accordance with the Declaration of Helsinki and approved by the institutional review boards (2019-064). Our diagnosis of the hepatic irAE is based on the liver function tests performed in each department. In the possible cases, liver biopsy and full-liver screening tests have performed to exclude both infectious and metabolic etiologies (including hepatitis A, B, C, or E; cytomegalovirus (CMV); Epstein–Barr virus; Wilson's disease; hemochromatosis; and other metabolic diseases) and autoantibody screening tests (including antinuclear and antimitochondrial antibody tests). Abdominal ultrasound or computed tomography was performed to exclude focal lesions in the liver or biliary tracts. The elevation of the liver enzymes that may be affected by drinking alcohol, infection, drug, and liver metastasis was also excluded. Forty patients were excluded because the liver enzyme elevations were caused by liver metastasis. Four patients were excluded because the liver enzyme elevations were caused by drug.  Figure 1 shows the flow chart of patients with hepatic irAE in this study.

### 2.2. Laboratory and Clinical Parameters

Clinical characteristics, including sex and age, were recorded. Laboratory tests, including aspartate aminotransferase (AST; normal range, 13–30 IU/L), alanine aminotransferase (ALT; normal range, male 10–42 IU/L, female 7–23 IU/L), total bilirubin (T-Bil, normal range, 0.4–1.5 mg/dL), alkaline phosphatase (ALP; normal range, 106–322 IU/L), gamma-glutamyltransferase (*γ*GTP; normal range, male 13–64 IU/L, female 9–32 IU/L), and prothrombin level (normal range, ≥70%), were evaluated. All patients with hepatic irAE were graded by the common toxicity criteria for adverse events (CTCAE) of the National Cancer Institute, version 4.0. The pattern of liver injury was classified as drug-induced liver injury (DILI) using the *R* value, which is defined as (ALT/ALT upper limit of normal (ULN))/(ALP/ALP ULN) [16–18]. The hepatocellular type is characterized by ALT ≥2 times ULN and *R* ≥ 5 times ULN; cholestatic type characterized by ALP ≥2 times ULN and *R* ≤ 2 times ULN; and mixed type characterized with ALT ≥2 times ULN, ALP ≥2 times ULN, and 2 times *R* < 5 times ULN.

### 2.3. Immune Checkpoint Inhibitors

The patients were treated with the following ICIs: (1) anti-CTLA-4 ipilimumab 3 mg/kg intravenously every 3 weeks; (2) anti-PD-1 nivolumab 240 mg/body intravenously every 2 weeks; (3) anti-PD-1 pembrolizumab 200 mg/body intravenously every 3 weeks; (4) a combination of anti-PD-1 nivolumab 240 mg/body + anti-CTLA-4 ipilimumab 1 mg/kg intravenously every 3 weeks (4 courses) followed by nivolumab 240 mg/body every 2 weeks; (5) anti-PD-L1 atezolizumab 1200 mg/body intravenously every 3 weeks; and (6) anti-PD-L1 durvalumab 10 mg/kg intravenously every 2 weeks. In 387 enrolled patients, 280 were treated with nivolumab, 75 with pembrolizumab, 8 with atezolizumab, 9 with durvalumab, 7 with ipilimumab, and 8 with a combination of nivolumab and ipilimumab.

The type of cancer was lung (146/387, 37.7%), followed by melanoma (86/387, 22.2%), head and neck (67/387, 17.3%), renal (53/387, 13.7%), stomach (27/387, 7.0%), and others (8/387, 2.1%). Anti-PD1 mAbs were used more frequently than anti-CTLA4 mAbs because anti-PD1 mAbs, especially nivolumab, could be used for most of these cancers. Fifty-six patients developed liver injury (all grade, 56/343, 16.3%), and severe liver injury was observed in 11 patients (grade ≥3, 11/343, 3.2%) according to the CTCAE system (ALT more than five times ULN). Among the 316 patients with anti-PD-1 (nivolumab or pembrolizumab), 49 patients developed liver injury (all grade, 49/316, 15.5%) and severe liver injury occurred in 7 patients (grade ≥3, 7/316, 2.2%). Among the 16 patients with anti-PD-L1 (atezolizumab or durvalumab), 1 patient developed liver injury (all grade, 1/16, 6.3%) and severe liver injury was not observed in any patients (grade ≥3, 0/16, 0%). Among the five patients with anti-CTLA-4 (ipilimumab), three developed liver injury (all grade, 3/5, 60.0%) and severe liver injury occurred in two patients (grade ≥3, 2/5, 40.0%). Among the six patients treated with both anti-CTLA-4 (ipilimumab) and anti-PD-1 (nivolumab), liver injury (all grade) and severe liver injury (grade ≥3) were observed in three patients (3/6, 50.0%) and two patients (2/6, 33.3%), respectively (Table 1).

### 2.4. Histological Evaluation

Four patients underwent liver biopsy, and liver specimens were fixed with 10% formaldehyde. Paraffin-embedded sections were prepared and subjected to hematoxylin and eosin staining and Masson trichrome staining. Immunostaining for multiple lymphocyte markers was performed using the following antibodies: CD3 (clone PS1, prediluted, Nichirei Biosciences, Tokyo, Japan), CD8 (clone 4B11, prediluted, Leica Biosystems, Nussloch, Germany), and CD20 (clone IgG2a, dilution 1 : 500, Dako Cytomation, Glostrup, Denmark). Sections were deparaffinized and rehydrated through xylene and ethanol. Antigen retrieval was performed by heating tissue sections at 100°C in 10 mM sodium citrate buffer (pH 6.0) (CD3), or 1 mM EDTA buffer (pH 8.0) (CD8) for 20 min in a microwave oven. Antigen retrieval was not performed in the CD20 immunostaining. Endogenous peroxidase activity was blocked by methanol containing 3% hydrogen peroxidase for 5 min. The sections were incubated with the primary antibodies at room temperature for 90 min, followed by staining with the secondary antibody. The sections were stained for 2.5–5.0 min with diaminobenzidine tetrahydrochloride (Nichirei Bioscience, Tokyo, Japan) and were then counterstained with hematoxylin (Thermo Fisher Scientific, Inc., Waltham, MA), dehydrated, and mounted. All biopsies were reviewed by a single liver pathologist without reference to clinical details.

### 2.5. Statistical Analysis

All statistical analyses were performed using JMP® 14 (SAS Institute Inc., Cary, NC). Sex, antinuclear antibody, and type of liver injury were compared using Fisher's exact test. The Wilcoxon rank-sum test was used for other univariate comparisons between the patient groups. A *p*-value less than 0.05 was considered statistically significant.

## 3. Results

### 3.1. Clinical Characteristics and Laboratory Test Results

Overall population characteristics are detailed in Table 1. In 56 patients with all-grade liver injury, the median age was 63 (range, 49–69) years and 25 patients were female (36.4%). The median duration from ICI initiation to liver injury was 45.5 days (range, 21–94), and the average duration was 87.4 days. Peak levels of laboratory tests included a median ALT 60 (range, 39–136) IU/L, AST 58 (range, 47–129) IU/L, ALP 471 (range, 263–857) IU/L, *γ*GTP 95.5 (range, 47–276) IU/L, and T-Bil 0.7 (range, 0.4–1.0) mg/dL. In the patients with all-grade liver injury, the hepatocellular type was only 19.6% (11/56), though the cholestatic or mixed-type injury was 60.7% (34/56). Prior existence of the extrahepatic irAEs did not significantly differ in grade ≤2 liver injury from that in grade ≥3 liver injury (grade 1 or grade 2, 6/45 (13.3%) vs. grade 3 or grade 4, 1/11 (9.1%), *p*=0.4749). In 11 patients with grade ≥3 liver injury, antinuclear antibodies were present in 9.0% (2/11) of patients. The median serum IgG level was 988 mg/dL, which is within the normal range (752–1216 mg/dL). The ratio of cholestatic or mixed-type liver injury tended to be higher in the anti-PD-1/PD-L1 mAbs group compared with the anti-CTLA-4 mAbs group or combination therapy group (4/7 (57.1%) in the anti-PD-1/PD-L1 mAbs group vs. 1/4 (25.0%) in the anti-CTLA-4 mAbs group, *p*=0.545). But other parameters as shown in Table 1 did not differ significantly between the anti-CTLA-4 mAbs group and anti-PD-1/PD-L1 mAbs group in this study.

### 3.2. Treatment

**Figure 2:**
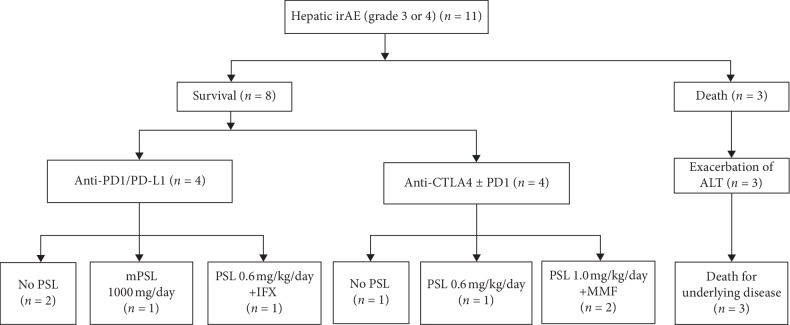
Flowchart of treatment of patients with >grade 3 liver injury. ALT, alanine aminotransferase; PD-1, programmed cell death 1; PD-L1, programmed cell death ligand 1; anti-CTLA-4, cytotoxic T-lymphocyte antigen 4; PSL, prednisolone; IFX, infliximab; MMF, mycophenolate mofetil.

The treatment of liver injury was chosen according to each patient's clinical features. Most patients with grade ≤2 liver injury improved spontaneously. One case of liver injury with biliary dysfunction was treated with ursodeoxycholic acid (UDCA) 600 mg/day, and another case (case 4) was treated with prednisolone 0.6 mg/kg/day. These two patients gradually improved. Treatments for the patients with grade ≥3 liver injury are summarized in Figure 2. Two patients improved spontaneously. One patient was treated with UDCA 600 mg/day. Two patients were treated with prednisolone 0.6 mg/kg and two patients with prednisolone 1.0 mg/kg. One patient was treated with UDCA 600 mg/day and methylprednisolone 1000 mg/day for 3 days. Details are shown in Case 1, below. In these prednisolone-treated patients, steroid-resistant liver injury was observed in three patients. Two patients were treated with mycophenolate mofetil 2000 mg/day, and one patient was treated with infliximab because he also suffered from checkpoint inhibitor-induced pneumonitis.

## 4. Case Series

**Figure 3:**
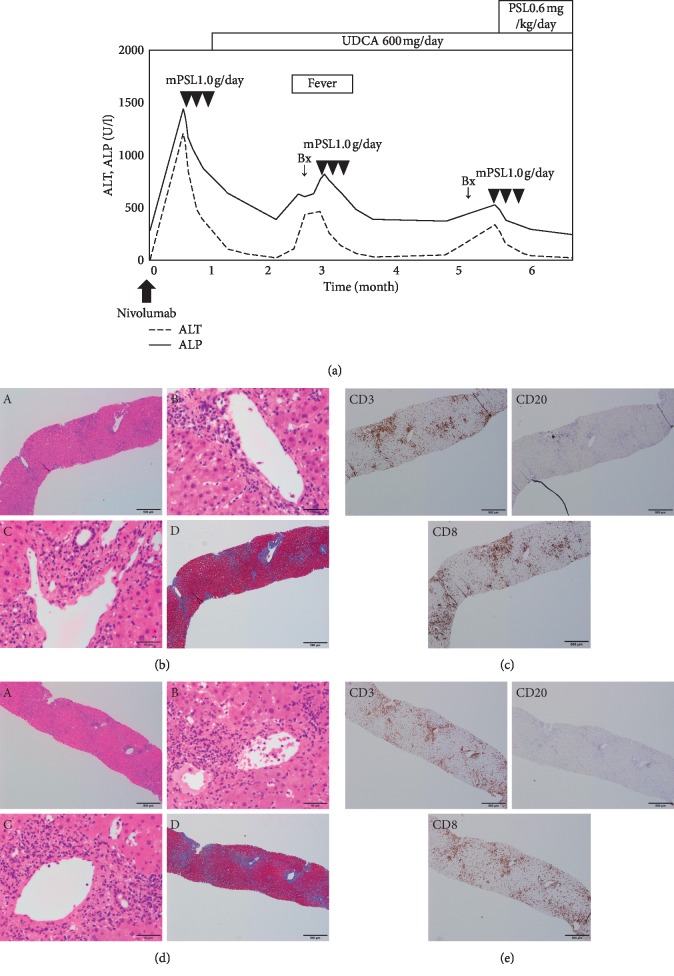
Chronological changes of the liver function tests and a summary of treatments in Case 1 (a). Microscopic images of HE staining, MT staining, and immunostaining from the first liver biopsy (b, c) and the second liver biopsy (d, e). (b, d) HE staining: (A) low-power field, (B) central vein area, and (C) portal area; MT staining: (D) low-power field. The liver parenchyma is mainly damaged with severe infiltration of lymphocytes. The first and second biopsy showed the development of fibrosis (b, d). (c, e) CD3, upper left panel; CD20, upper right panel; CD8, lower left panel. The number of CD3 or CD8 positive lymphocytes is markedly higher than that of CD20 positive lymphocytes. ALT, alanine aminotransferase; ALP, alkaline phosphatase; UDCA, ursodeoxycholic acid; Bx, biopsy; PSL, prednisolone; mPSL, methylprednisolone; HE, hematoxylin and eosin; MT, Masson trichrome; CD, cluster of differentiation.

**Figure 4:**
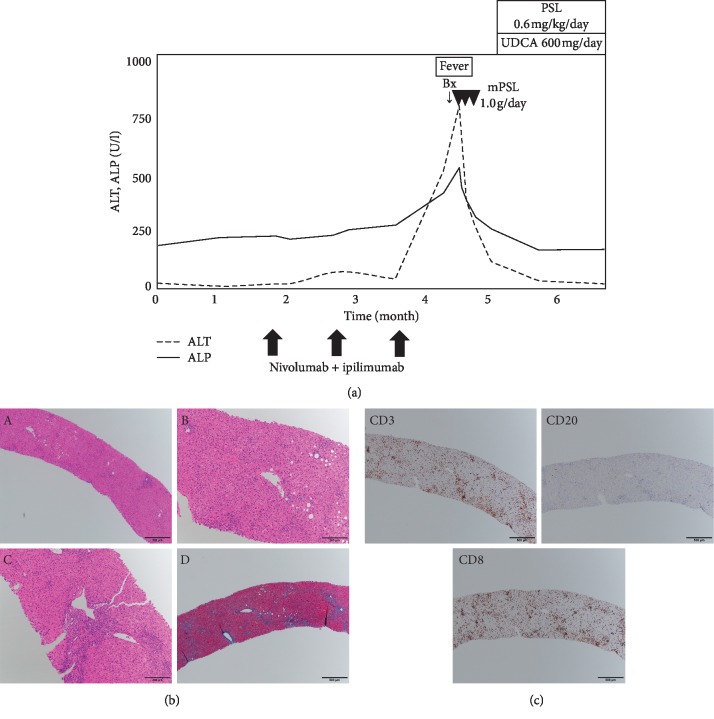
Chronological changes of the liver function tests and a summary of treatments in Case 2 (a). Microscopic images of HE staining, MT staining, and immunostaining from the liver biopsy (b, c). HE staining: (A) low-power field, (B) central vein area, and (C) portal area; MT staining: (D) low-power field. The liver parenchyma is mainly damaged with moderate infiltration of lymphocytes. CD3, upper left panel; CD20, upper right panel; CD8, lower left panel. The number of CD3 or CD8 positive lymphocytes is markedly higher than CD20 positive lymphocytes. ALT, alanine aminotransferase; ALP, alkaline phosphatase; UDCA, ursodeoxycholic acid; Bx, biopsy; PSL, prednisolone; mPSL, methylprednisolone; HE, hematoxylin and eosin; MT, Masson trichrome; CD, cluster of differentiation.

**Figure 5:**
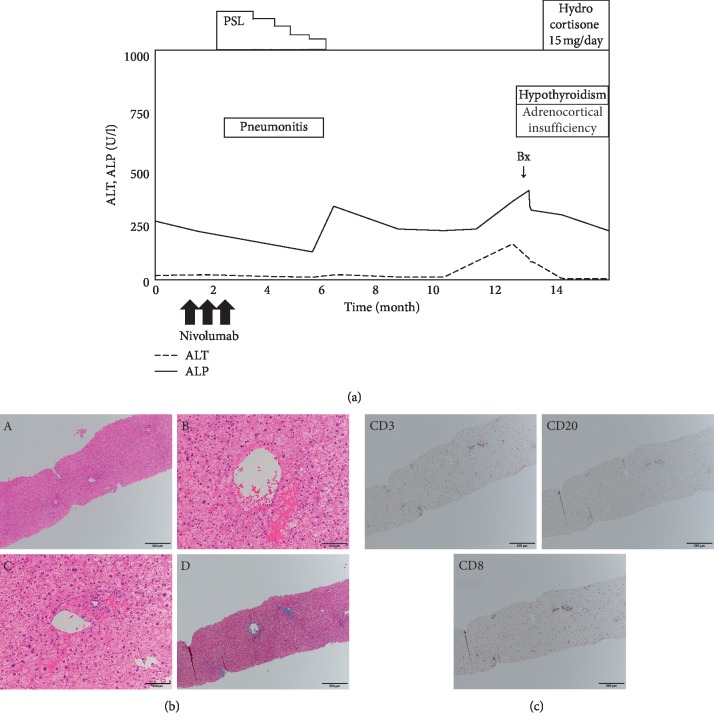
Chronological changes of the liver function tests and a summary of treatments in Case 3 (a). Microscopic images of HE staining, MT staining, and immunostaining from the liver biopsy (b, c). HE staining: (A) low-power field, (B) central vein area, and (C) portal area; MT staining: (D) low-power field. The liver parenchyma is mainly damaged with moderate infiltration of lymphocytes. CD3, upper left panel; CD20, upper right panel; CD8, lower left panel. The number of CD3 or CD8 positive lymphocytes is markedly higher than CD20 positive lymphocytes. ALT, alanine aminotransferase; ALP, alkaline phosphatase; UDCA, ursodeoxycholic acid; Bx, biopsy; PSL, prednisolone; mPSL, methylprednisolone; HE, hematoxylin and eosin; MT, Masson trichrome; CD, cluster of differentiation.

**Figure 6:**
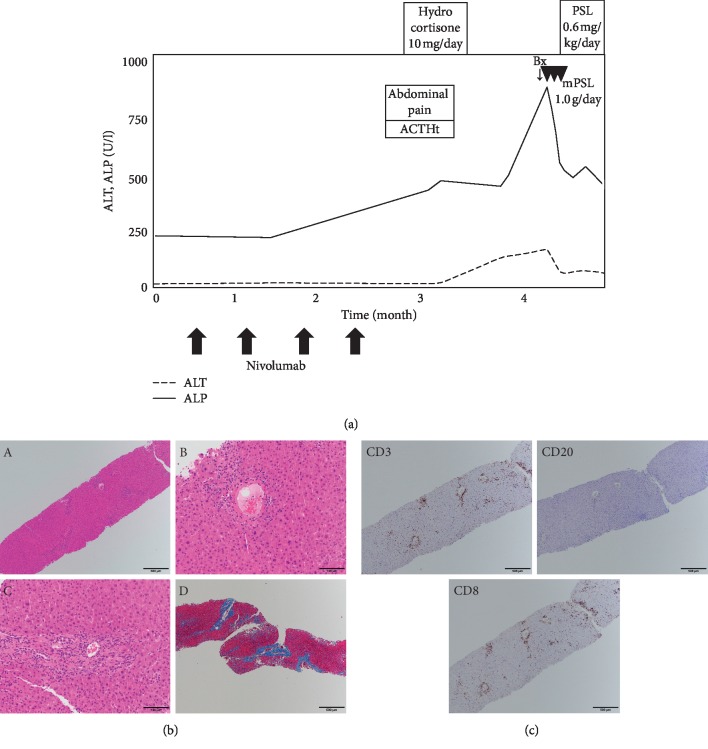
Chronological changes of the liver function tests and a summary of treatments in Case 4 (a). Microscopic images of HE staining, MT staining, and immunostaining from the liver biopsy (b, c). HE staining: (A) low-power field, (B) central vein area, and (C) portal area; MT staining: (D) low-power field. The portal area is mainly damaged with mild infiltration of lymphocytes. Infiltration of lymphocytes is also seen in the bile ducts. CD3, upper left panel; CD20, upper right panel; CD8, lower left panel. The number of CD3 or CD8 positive lymphocytes is markedly higher than CD20 positive lymphocytes. ALT, alanine aminotransferase; ALP, alkaline phosphatase; UDCA, ursodeoxycholic acid; Bx, biopsy; PSL, prednisolone; mPSL, methylprednisolone; HYD, hydrocortisone; ACTH, adrenocorticotropic hormone; HE, hematoxylin and eosin; MT, Masson trichrome; CD, cluster of differentiation.

Four patients who had undergone liver biopsies for the hepatic irAE were reviewed retrospectively. The clinical courses, blood tests, and pathological findings of cases 1–4 are summarized in Figures 3–6, respectively. Clinical features of 4 patients are summarized in Table 2.

### 4.1. Case 1

A 75-year-old male with hypopharyngeal carcinoma was treated initially with surgical resections. After further disease progression in the lung, nivolumab was administered (240 mg/body intravenously, planned for every 2 weeks). A baseline CT scan demonstrated no liver metastasis. Fourteen days after a single infusion of nivolumab, the liver enzymes were markedly elevated (ALP 1453 U/L, *γ*GTP 751 U/L, AST 1180 U/L, and ALT 1220 U/L) (Figure 3(a)). General fatigue and loss of appetite appeared, but there was no fever. A CT scan did not demonstrate focal lesions either in the liver or biliary tracts. Full-liver screening tests excluded both infectious and metabolic etiologies (including hepatitis A, B, C, or E; cytomegalovirus (CMV); Epstein–Barr virus and adenovirus infection; Wilson's disease; hemochromatosis; and other metabolic diseases) and autoantibody screening tests (including antinuclear and antimitochondrial antibody tests) were negative. Serum immunoglobulin revealed hypogammaglobulinemia (707 mg/dL). The medications were not changed except for the induction of nivolumab. Liver biopsy was not performed because of ascites, and methylprednisolone (1000 mg/day) was immediately administered for 3 days. Because the possibility of infection could not be denied, and his liver enzymes improved rapidly, prednisolone was not administered orally. UDCA 600 mg/day was commenced, and transaminases decreased gradually. After 2 months from the first administration of nivolumab, fever, general fatigue, and liver enzyme elevation appeared. Though there was no fever at the first admission, we suspected the recurrence of the hepatic irAE and performed a diagnostic liver biopsy. Severe hepatitis with lobular inflammation and moderate fibrosis without granulomatous inflammation were observed on HE staining (Figure 3(b)). We performed immunostaining to evaluate immune cell infiltration, including T lymphocytes (CD3+), cytotoxic T lymphocytes (CD8+), and B cells (CD20+). CD3+ and CD8+ lymphocytes were predominantly observed, while fewer CD20+ lymphocytes were detected (Figure 3(c)). These findings suggested an acute or subacute response of the hepatic irAE. Methylprednisolone 1000 mg/day was administered for 3 days again, and the liver enzymes improved immediately. Oral prednisolone was not administered because the general condition of the patient was very poor. After 2 months, the liver enzymes were elevated again without any symptoms. Although 6 months had passed since the first administration of nivolumab, the hepatic irAE was suspected and a diagnostic liver biopsy was performed again. The histological findings were the same as before (Figures 3(d) and 3(e)). Methylprednisolone 1000 mg/day for 3 days and oral prednisolone (0.6 mg/kg) were commenced sequentially. The liver enzymes have not shown any exacerbation for more than a year.

### 4.2. Case 2

A 52-year-old male with renal cell carcinoma concomitant with bone and lung metastasis was treated initially with surgical resections of the primary site. One month later, nivolumab (240 mg/body intravenously, planned for every 2 weeks) and ipilimumab (1 mg/kg intravenously, planned for every 3 weeks) were administered as additional therapy. The liver enzymes began to worsen (ALP 517 U/L, *γ*GTP 101 U/L, AST 471 U/L, and ALT 780 U/L), and general fatigue and fever appeared 3 months after ICI initiation (Figure 4(a)). Liver screening tests were unremarkable, similar to Case 1. Antinuclear antibody (ANA) was negative, and IgG was within the normal range (1216 mg/dL). The medications were not changed, aside from nivolumab and ipilimumab. A CT scan did not demonstrate any focal lesions in the liver and biliary tracts. A diagnostic liver biopsy was performed, and methylprednisolone 1000 mg/day was immediately administered for 3 days. After the treatment with methylprednisolone, prednisolone 0.6 mg/kg and UDCA 600 mg/day were commenced orally. Liver biopsy demonstrated moderate hepatitis with lobular inflammatory activity, but without fibrosis and granulomatous inflammation on HE staining (Figure 4(b)). Immunostaining revealed predominantly CD3+ and CD8+ lymphocytes, while fewer CD20+ lymphocytes were detected (Figure 4(c)). These findings suggested an acute response of the hepatic irAE. The liver enzymes improved and have not been exacerbated.

### 4.3. Case 3

A 72-year-old male with esophageal cancer was treated initially with surgical resection. Four months later, nivolumab (240 mg/kg intravenously, planned for every 2 weeks) was administered as adjuvant therapy. After three doses of nivolumab, interstitial pneumonitis occurred as an irAE. Prednisolone 1.0 mg/kg/day was commenced, with rapid clinical improvement (Figure 5(a)). Prednisolone was tapered gradually and was ceased totally after 4 months. Pneumonitis-related symptoms were resolved completely. Two months after discontinuing prednisolone, blood tests incidentally revealed an increase of eosinophils (31.0%, absolute total eosinophils 1078/*μ*L). This finding was carefully observed because of no concomitant symptoms. Six months after the discontinuation of prednisolone, liver enzymes began to worsen (ALP 342 U/L, *γ*-GTP 124 U/L, AST 153 U/L, and ALT 155 U/L). ANA was negative, and IgG was 1923 mg/dL. Liver screening tests were unremarkable, as with Case 1, while thyroid function tests demonstrated hypothyroidism (TSH 104.6 *μ*IU/mL, fT4 0.2 ng/dL). We performed a diagnostic liver biopsy because hepatic irAE or hypereosinophilic syndrome (HES) could be the cause of liver enzyme elevations. Adrenocortical insufficiency as an irAE also could not be denied (eosinophil 31.0%, Na 131 mmol/L, ACTH 14.0 pg/mL, and cortisol 6.2 *μ*g/dL), so hydrocortisone 15 mg/day was started before levothyroxine administration. After the diagnosis of adrenocortical insufficiency by endocrinological tests (high ACTH and low cortisol levels before and after the corticotropin releasing hormone stimulation test and ACTH stimulation test), levothyroxine 25 *μ*g/day was commenced. Liver biopsy demonstrated mild hepatitis with portal inflammatory activity without infiltration of eosinophils and fibrosis on HE staining (Figure 5(b)). Immunostaining revealed predominantly CD3+ and CD8+ lymphocytes, while fewer CD20+ lymphocytes were detected (Figure 5(c)). These findings suggested an acute response of the hepatic irAE. The liver enzymes improved immediately and did not worsen again.

### 4.4. Case 4

A 58-year-old male with gastric cancer was treated initially with surgical resections. After disease progression with peritoneum invasion, nivolumab was administered (240 mg/body intravenously, planned for every 2 weeks). A baseline CT scan demonstrated no lesions in the liver or biliary tracts. Two months after the first administration of nivolumab, abdominal pain and elevation of ACTH appeared (ACTH 99.2 pg/mL and cortisol 36.9 *μ*g/dL). Adrenocortical insufficiency as an irAE was highly suspected, and hydrocortisone 10 mg/day was commenced. However, endocrinological tests denied adrenocortical insufficiency (2 days after the previous tests, ACTH 12.1 pg/mL and cortisol 19.0 *μ*g/dL), and the abdominal pain disappeared. Afterwards, the liver enzymes began to worsen (ALP 861 U/L, *γ*GTP 297 U/L, AST 199 U/L, and ALT 163 U/L) (Figure 6(a)). Liver screening tests were unremarkable, similar to Case 1. ANA was negative, and IgG was within the normal range (910 mg/dL). The medication was not changed, aside from discontinuation of nivolumab. A CT scan demonstrated intrahepatic and extrahepatic biliary dilatations and stenosis of the lower bile duct, upper bile duct, and right hepatic duct. A diagnostic liver biopsy was performed, and methylprednisolone 1000 mg/day was administered for 3 days, followed by oral prednisolone 0.6 mg/kg/day and UDCA 600 mg/day. Liver biopsy demonstrated mild hepatitis with infiltration of lymphocytes in the liver parenchyma, portal area, and bile duct on HE staining (Figure 6(b)). Immunostaining revealed predominantly CD3+ and CD8+ lymphocytes, while fewer CD20+ lymphocytes were detected. CD3+ and CD8+ lymphocytes infiltrated in the bile ducts (Figure 6(c)). These findings suggested an acute response of the hepatic irAE. The liver enzymes gradually improved and did not worsen again.

### 4.5. Human Leukocyte Antigens

Human leukocyte antigen (HLA)‐typing was performed in the four cases described above (Table 2), and the HLA associated with AIH was not detected. A : 24 : 02, A *∗* 33 : 03, and DRB1 *∗* 09 : 01 were generally matched among the patients, but these HLAs are common in the Japanese population.

## 5. Discussion

### 5.1. Clinical Features of Hepatic irAE

Here, we examined a clinical dataset of patients with ICI-induced liver injury, i.e., hepatic irAE. The hepatic irAE presents various patterns of clinical course. Some patients improved spontaneously, while others required immune suppressants.

Liver injury was observed in 16.3% of patients with ICI administration, and severe liver injury (grade ≥3) was diagnosed in only 3.2%. Compared with previous reports [10–12], the incidence of all-grade liver injury was higher and that of grade ≥3 liver injury was similar in our cohort. In this study, the extent of ALT and ALP elevation was heterogeneous, not like classical AIH. Furthermore, cholestatic and mixed-type liver injuries were more frequent than the hepatocellular type, and only one case showed bile duct dilation on imaging tests (Case 4). This result is reasonable because the hepatic irAEs with abnormal image findings in the bile ducts have been reported (acute cholangitis [19–21] and primary sclerosing cholangitis [22]), but their incidence is rare. These findings suggest that the bile duct disorder on the hepatic irAEs might occur at the microscopic level. Biliary epithelial cells express PD-1 ligands (PD-L1 and PD-L2) [23] but do not express CTLA-4 ligands (B7 (CD80 or CD86) molecules) [24]. In our patients, cholestatic or mixed-type liver injury was more frequent in the anti-PD-1 and/or PD-L1 group compared with the anti-CTLA-4 group. In addition, acute cholangitis was only reported in patients treated with anti-PD-1 mAbs [19–21]. These findings suggest that the inhibition of the immune checkpoint pathway by anti-PD-1 or PD-L1 mAbs may be a cause of biliary disorder in the hepatic irAE. However, careful attention to ethnicity bias is needed because all the patients in this study were Japanese.

The hepatic irAE typically occurs between 6 and 14 weeks after the initiation of ICIs, as previously reported [25]. The hepatic irAE usually resolves within 4–6 weeks with appropriate treatment but is prolonged occasionally. The terminal half-life (t1/2) of ipilimumab, nivolumab, and pembrolizumab has been reported to be 14–23 days [26], 12–20 days [27], and 14–22 days [28], respectively. In Case 1, the liver function tests exacerbated three times within a half year, although nivolumab was administered only one time. In Case 3, liver injury occurred 10 months after nivolumab therapy. The previous report shows that prolonged nivolumab binding to T lymphocytes was detected more than 20 weeks after the last infusion, regardless of the total number of nivolumab infusions [29]. The persistence of liver injury in Case 1 and Case 3 may be explained by prolonged nivolumab binding. Therefore, we should carefully follow-up even after the administration of ICIs is finished.

### 5.2. Histopathological Features of Hepatic irAE

The hepatic irAE is thought unlikely to present the characteristic features of classical AIH, such as plasma cell infiltration, rosette formation, and interface hepatitis. A previous report showed that the hepatic irAE was predominantly characterized by lobular hepatitis with CD3+ or CD8+ lymphocyte infiltration, but not with CD20+ lymphocytes [30]. In the current study, the histological findings were almost the same as those of previous reports, but granulomatous hepatitis with fibrin deposition was not observed. Histological differences were not evident between anti-PD-1/PD-L1 mAbs group and anti-CTLA-4 mAbs group. Liver biopsy in Case 1 showed severe hepatitis with lobular inflammatory activity, although transaminase was about 400 IU/l. Moderate fibrosis was also observed in Case 1 even though only 2 months passed from the first administration of nivolumab. These findings suggest that the hepatic irAE can induce rapid fibrosis.

### 5.3. The Treatments for Hepatic irAE

The management of irAEs is important because the development of irAEs deteriorate survival of patients with various carcinomas treated with ICIs [31, 32]. The current recommendation for grade ≥2 liver injury, according to the CTCAE system, is corticosteroid therapy at a dose of 1–2 mg/kg/day [33]. In the event of grade ≤2 liver injury, checkpoint inhibitor should be withheld in our hospital. If worsening or no improvement occurs, the patients are started to treat with prednisolone 0.6–1.0 mg/kg/day orally. If T-Bil is over 3.0 mg/dl or PT% is under 60%, the patients are treated with methylprednisolone 1000 mg/day for 3 days initially. Most patients with grade 1 or 2 liver injury recovered naturally without corticosteroid therapy, while patients with liver injury involving the bile duct disorder required medications (UDCA or prednisolone). Among the patients with grade 3 or grade 4 liver injury, three patients did not receive prednisolone therapy. Two patients were treated with prednisolone less than 1.0 mg/kg (0.6 mg/kg), and their liver function tests were improved. These findings suggest that the demanding dose of steroids for the hepatic irAE could be less than the recommended dose in the CTCAE system. Because steroids increase the risk of serious infections among patients receiving ICIs [34], and the influence of corticosteroids on antitumor effects remain controversial [35, 36], reduced steroid dosage can be considered in patients with low-grade liver injury. Steroids are useful for many patients with hepatic irAE, but steroids and other immunosuppressants may have a limited role in severe injury with biliary involvement [20, 37]. UDCA is known to be effective for cholestatic liver disease like primary biliary cholangitis and is used in some cases of drug-induced liver injury with cholestasis. In our hospital, UDCA is used in cases with liver injury involving the bile duct disorder and was also used in cases with severe liver injury in combination with steroids. Some cases showed the possible usefulness of UDCA treatment for the hepatic irAEs with biliary involvement. Infliximab is an antitumor necrosis factor monoclonal antibody and is reported to be useful for the management of severe ICI-induced colitis and pneumonitis. However, infliximab has not yet been reported as a management option for the hepatic irAE because infliximab has the possibility of worsening liver function [38]. However, infliximab has been used for the management of refractory autoimmune hepatitis [39]. In the current study, infliximab was used in one case because the patient had ICI-induced pneumonitis with hepatic irAE, and his liver function tests gradually improved. Infliximab may be effective for the hepatic irAE, but further studies are necessary for evaluating the benefit of infliximab.

### 5.4. HLA Types and Hepatic irAE

A previous report evaluating the relationship between irAEs and HLA types showed that pruritus and colitis were associated with HLA-DRB1 *∗* 11 : 01 and HLA-DQB1 *∗* 03 : 01, respectively [40]. However, the association between hepatic irAEs and HLA types has not been demonstrated yet. It has been reported that AIH is associated with HLA DRB1 *∗* 03 : 01, DRB1 *∗* 04 : 01, DRB1 *∗* 04 : 05, and DRB1 *∗* 15 : 01 [41, 42]; however, the HLA types of our patients did not show any association with risk alleles of irAE-related pruritus and colitis or AIH (Table 3).

Our study has some limitations. Firstly, liver biopsy is desirable to diagnose the hepatic irAE precisely; however, our data were collected retrospectively, and biopsy was not performed in all cases. Secondly, this was a single-center investigation, and the number of patients who were treated with anti-CTLA4 mAbs was small.

In conclusion, this study showed that the hepatic irAE took diverse clinical features (hepatocellular type or cholestatic type and absence or presence of fever), and the management of hepatic irAEs in Japanese patients is similar to the previous report [43]. We also showed that ICIs could cause microscopic biliary disorder without any abnormal image finding, and we demonstrated the possible usefulness of UDCA treatment for the hepatic irAEs with biliary involvement. The hepatic irAE is generally treated without histological assessment; however, we recommend liver biopsy to diagnose the hepatic irAE precisely and to choose an appropriate treatment.

We assume that the number of hepatic irAE cases will increase because the indication for ICIs is expanding. Further studies are required to clarify the mechanisms and predictive factors of the hepatic irAE.

## Figures and Tables

**Table 1:** Comparison of patients with grade ≤2 liver injury and grade ≥3 liver injury induced by ICIs.

Characteristic	All grade	Grade 1 or 2	Grade 3 or 4	*p* value
The number of patients	56/343 (16.3%)	45/56 (80.4%)	11/56 (19.6%)	
Median age, years	63 (49–69)	63.0 (50–70)	58.0 (48.0–67.0)	0.4327
Sex, male	31 (63.6%)	24/45 (53.3%)	7/11 (63.6%)	0.7373
Previous extrahepatic irAEs	7/56 (12.5%)	6/45 (13.3%)	1/11 (9.1%)	0.4749
Duration of ICIs until liver injury, days	45.5 (21–94)	56 (26–134)	26 (20–56)	0.1116
T-Bil (mg/dl)	0.7 (0.4–1.0)	0.6 (0.4–0.8)	0.8 (0.5–1.3)	0.0871
AST (IU/l)	60 (39–136)	46 (37–87)	321 (101–1180)	<0.001
ALT (IU/l)	58 (47–129)	53 (45–97)	372 (228–780)	<0.001
ALP (IU/l)	471 (263–857)	431 (242–783)	811 (436–1453)	0.0620
*γ*GTP (IU/l)	95.5 (47–276)	79 (38–250)	187 (90–630)	0.0391
Hepatocellular type	11/56 (19.6%)	5/45 (11.1%)	6/11 (54.5%)	0.0041
Cholestatic or mixed type	34/56 (60.7%)	29/45 (64.4%)	5/11 (45.5%)	0.3101

ICIs: immune checkpoint inhibitors; irAEs: immune-related adverse events; T-Bil: total bilirubin; AST: aspartate aminotransferase; ALT: alanine aminotransferase; ALP: alkaline phosphatase; *γ*GTP: gamma-glutamyltransferase.

**Table 2:** Clinical features of four patients with hepatic irAE.

Case	Age	Sex	Primary lesion	ICIs	Duration of ICIs until liver injury, days	Clinical symptoms	The pattern of liver injury	Histologic pattern of liver injury	Therapy
1	75	M	Hypopharyngeal	NIVO	14	Loss of appetite	Hepatocellular	Panlobular hepatitis Moderate fibrosis	Steroid + UDCA
2	52	M	Renal	NIVO + IPI	63	Fever	Hepatocellular	Panlobular hepatitis	Steroid + UDCA
3	72	M	Esophageal	NIVO	360	No symptoms	Mixed	Portal >zone 3 hepatitis	Steroid
4	58	M	Gastric	NIVO	56	Abdominal pain	Cholestatic	Portal hepatitis cholangitis	Steroid + UDCA

irAEs: immune-related adverse events; ICIs: immune checkpoint inhibitors; NIVO: nivolumab; IPI: ipilimumab; UDCA: ursodeoxycholic acid.

**Table 3:** HLA of 4 patients with hepatotoxicity induced by ICIs.

	A1	A2	B1	B2	C1	C2	DR1	DR2
Case 1	A : 24 : 02	A *∗* 33 : 03	B *∗* 44 : 03	B *∗* 52 : 01	C *∗* 12 : 02	C *∗* 14 : 03	DRB1 *∗* 08 : 03	DRB1 *∗* 15 : 02
Case 2	A : 26 : 01	A:33 : 03	B *∗* 40 : 06	B *∗* 08 : 01	C *∗* 08 : 01	C *∗* 14 : 03	DRB1 *∗* 08 : 03	DRB1 *∗* 09 : 01
Case 3	A *∗* 11 : 01s	A *∗* 24 : 02	B *∗* 52 : 01	B *∗* 55 : 02	C *∗* 01 : 02	C *∗* 12 : 02	DRB1 *∗* 09 : 01	DRB1 *∗* 15 : 02
Case 4	A : 24 : 02	A *∗* 33 : 03	B *∗* 40 : 02	B *∗* 58 : 01	C *∗* 03 : 02	C *∗* 03 : 04	DRB1 *∗* 09 : 01	DRB1 *∗* 12 : 01

## Data Availability

The clinical data used to support the findings of this study are included within the article.
